# How Much Physiotherapy, Chiropractic, and Osteopathy Care Do Compensated Australian Workers with Low Back Pain Receive? A Retrospective Cohort Study

**DOI:** 10.1007/s10926-024-10202-1

**Published:** 2024-05-18

**Authors:** Michael Di Donato, Shannon Gray, Luke R. Sheehan, Rachelle Buchbinder, Ross Iles, Alex Collie

**Affiliations:** School of Public Health and Preventive Medicine, Monash University, 553 St Kilda Road, Melbourne VIC 3000, Australia

**Keywords:** Workers, Compensation, Low back pain, Physical therapy

## Abstract

**Objectives:**

To identify the prevalence and frequency of physiotherapy, chiropractic, and/or osteopathy care in Australians with workers’ compensation claims for low back pain (LBP).

**Methods:**

We included workers with accepted workers’ compensation claims longer than 2 weeks from the Australian states of Victoria, Queensland, South Australia, and Western Australia. Workers were grouped by whether they attended physiotherapy, chiropractic, and/or osteopathy in the first 2 years of their claim. Descriptive statistics and logistic regression were used to describe differences between groups. Descriptive statistics and negative binomial regression were used to describe differences in the number of attendances in each group.

**Results:**

Most workers had at least one physical therapy attendance during the period of their claim (n = 23,619, 82.0%). Worker state, socioeconomic status, and remoteness were the largest contributing factors to likelihood of physical therapy attendance. Most workers only attended physiotherapy (n = 21,035, 89.1%, median of 13 times). Far fewer only attended chiropractic (n = 528, 2.2%, median of 8 times) or only osteopathy (n = 296, 1.3%, median of 10 times), while 1,750 (7.5%) attended for care with more than one type of physical therapy (median of 31 times).

**Conclusion:**

Most Australian workers with workers’ compensation time loss claims for LBP attend physiotherapy at least once during their claims. State of claim is the strongest predictor of which physical therapy profession they attend, possibly due to regional availability. Workers who see a physiotherapist have significantly more attendances. Future research should explore the relationship between these patterns of care and claimant outcomes, including work disability duration.

**Supplementary Information:**

The online version contains supplementary material available at 10.1007/s10926-024-10202-1.

## Introduction

Low back pain is a leading cause of disability globally [1, 2]. Many health professionals including physiotherapists, chiropractors, and osteopaths can provide many of the recommended treatments for low back pain [3, 4]. However, the more specific therapeutic choices and modes of delivery may vary by clinician training and profession-specific guidelines [5–8], and these may be supported by varying levels of evidence [7, 9].

Workers’ compensation schemes fund reasonable and necessary health care and rehabilitation services for individuals who are unable to work due to work-related conditions, including low back pain [10, 11]. Funding for services constitutes approximately one quarter of workers’ compensation scheme expenditure in Australia [12]. While estimates vary, physical therapy contributes twenty to thirty percent of these healthcare costs [13–15]. For example, the workers’ compensation scheme in Western Australia (Australia’s fourth most populous state) funded nearly $16 million (AUD) in physiotherapy, chiropractic, and osteopathy services in 2020–21 [13].

While estimates of physical therapy patterns of care are available, these are either not specific to the workers’ compensation sector or are from single Australian jurisdictions or outside Australia [13, 14, 16–21]. Furthermore, the determinants of seeking physical therapy in compensated workers are unclear [22]. Understanding patterns of physical therapy care may be particularly relevant given that the type of treating provider can impact time to return to work [23]. We, therefore, sought to answer the following research questions: (1) what proportion of Australian workers with accepted workers’ compensation time loss claims for low back pain attend physiotherapists, chiropractors and/or osteopaths for physical therapy?; (2) how frequently do they attend?; and (3) what are the determinants of these attendances and their frequency?

## Methods

### Setting

There are 11 Australian workers’ compensation schemes, 1 in each state (n = 6) and territory (n = 2), as well as 3 national schemes [10, 24]. Each scheme provides funding for wage replacement and “reasonable and necessary” health care, including physiotherapy, chiropractic, and osteopath services. Physiotherapists, chiropractors, and osteopaths in Australia are primary health care clinicians and workers do not require a referral to attend for care. Workers can usually select their own health care practitioner, but most jurisdictions require health care providers to be registered with the workers’ compensation scheme insurer. There are variations in policies between schemes [24]. There are three notable macro-level variations relevant to this study. First, the Victorian and South Australian schemes apply a 10-business day (2 week) excess period in which the employer funds an injured workers’ wage replacement. Second, there is also a medical excess amount of approximately AUD$700 in Victoria, which must be exceeded before the scheme will fund health care. Third, the limit on total health care expenditure varies. In Victoria, South Australia, and Queensland, there was no monetary limit on health care funding during the study period, whereas there was a limit of approximately AUD$60,000 total health care costs per claim in Western Australia [24].

### Data Sources

This study utilizes the Multi-Jurisdiction Workers’ Compensation Database [25]. The database includes claims and payments data from several Australian workers’ compensation jurisdictions for musculoskeletal conditions including low back pain. Claims data include information about the claimant such as claim acceptance date, age, sex, and occupation. Payment data include the type, date, and cost of all health care services for each claim. Data from all jurisdictions have previously been harmonized in a systematic and rigorous process to allow inter-jurisdictional analyses and comparisons in a process documented elsewhere [25].

We included physical therapy services provided by either a physiotherapist, chiropractor, and/or osteopath from Victoria, Queensland, South Australia, and Western Australia. We included these jurisdictions as services data were sufficiently detailed to identify and harmonize physical therapy services. These jurisdictions include approximately two thirds (7.5 million people) of the Australian labor force [26]. Any physical therapy service from 30 days before to 730 days (i.e., 2 years) after the claim acceptance date was included. We included services provided 30 days prior to claim acceptance as these services are also covered in an accepted claim. We included services up to 2 years from claim acceptance to standardize health care limits between workers’ compensation schemes. We excluded services that did not directly include patient care, i.e., writing reports and other paperwork, cancelations, and travel. To avoid inflating counts, we only included one visit per profession on a given date, i.e., if there was an individual and group session for the same therapist on the same day, we only included the individual session. Finally, it was possible that a worker could have more than one claim. However, we handled each claim as an individual period of care, referring to people as “workers”.

### Sample

We included all workers aged between 15 and 80 years with accepted time loss claims for low back pain in Victoria, Queensland, South Australia, and Western Australia accepted between 1 July 2011 and 30 June 2015. Low back pain was defined using the Type of Occurrence Classification System (TOOCS) with sample criteria available in the supplementary materials [27]. To control for excess period differences between jurisdictions, we included claims from Victoria and South Australia with any wage replacement payments and only claims from Queensland and Western Australia with at least 2 weeks’ wage replacement, as per our prior studies [11, 28, 29].

### Outcome Variables

Outcomes were (1) the proportion of workers that attended physiotherapists, chiropractors, and/or osteopaths and (2) the total number of attendances at each type of therapist per worker.

### Covariates

Several covariates were available and included in analyses: sex (male, female), age group (categorized as 15–25, 26–35, 36–45, 46–55, 56 + years), occupation (categorized using the Australia and New Zealand Standard Classification of Occupations), jurisdiction (state of claim), socioeconomic status, and remoteness. Socioeconomic status was identified via Index of Relative Socioeconomic Advantage and Disadvantage quintiles, matched to a worker’s residential postcode [30]. The middle three quintiles were collapsed for explanatory parsimony. Remoteness was identified by the Australian Remoteness Index for Areas, also matched to a worker’s residential postcode [31]. Due to small sample sizes, Inner Regional and Outer Regional Australia, and Remote and Very Remote Australia were collapsed into Regional Australia and Remote Australia, respectively. Total cumulative wage replacement duration (measured in weeks) was also available and included in analyses where appropriate, as described in the following section. Partial or full return to work was possible in this sample of workers but was challenging to reliably measure with the available data, hence the use of cumulative weeks’ wage replacement.

### Analysis

We identified the number of workers who attended for physical therapy at least once and the number of workers who attended each type of therapist (physiotherapist, chiropractor, osteopath). Individual and group physiotherapy sessions were described separately but were included together in subsequent calculations. Chiropractor and osteopath group sessions were not available in the data.

We then used descriptive statistics and binary logistic regression to compare workers that attended at least one physical therapy session versus those who did not attend any. The binary logistic regression model was adjusted for sex, age group, occupation, jurisdiction, socioeconomic status, and remoteness. Covariates were selected as factors previously identified as relevant to service use. Workers were then categorized into one of four groups: attendances solely with physiotherapists (i.e., physiotherapist only), chiropractors (chiropractor only) or osteopaths (osteopath only) or attendances to more than one of these professions (multiple professions). We used descriptive statistics to compare group sizes for all available covariates. A multinomial logistic regression model again adjusting for all available covariates was then used to compare the likelihood of being in the chiropractor only, osteopath only, or multiple professions groups compared to the physiotherapist only group. The output of logistic regression models was reported as odds ratios (OR) with 99% confidence intervals and statistical significance was set at p < 0.01.

Finally, we used descriptive statistics (median and inter-quartile range) and negative binomial regression to compare differences in the total number of attendances between each of the four groups. We produced two negative binomial models. The first adjusted for all covariates as per previous models (model 1). Given we were measuring the total number of visits, the second model (model 2) also adjusted for the cumulative total weeks of wage replacement as previous literature has identified a correlation between time loss duration and total number of services [32]. Output from negative binomial models was reported as incidence rate ratios (IRR) and statistical significance was set at p < 0.01.

Workers with missing data were excluded from statistical analyses. Statistical models with missing data handled through multiple imputation by chained equations were also performed and are available in the supplementary materials for comparison. Analyses were performed in RStudio using R 4.2.2 [33]. A number of packages were used and are listed in the supplementary materials. This study was approved by the Monash University Human Research Ethics Committee (Project ID 17267, November 2018).

## Results

The sample included 28,819 workers, with most workers (N = 23,619, 82.0%) attending for physical therapy at least once (see Table 1). A total of 596,432 attendances were included, of which the majority were physiotherapy attendances (N = 559,882, 93.9%). Most workers attended physiotherapists (N = 22,769, 89.5% of those receiving services), while fewer workers attended chiropractors (N = 1,808, 7.1%) and osteopaths (N = 873, 3.4%). The majority of physiotherapist attendances were individual sessions (N = 511,579, 91.4%).

**Table 1:** Number and percentage of workers and physical therapy attendances

	N (%) workers	Attendances		
		N (%) attendances	N (%) type of attendance	
Workers who attended for physical therapy at least once				
No attendance	5,200 (18.0)	−	−	−
Attendance to at least one of physiotherapist, chiropractor, and/or osteopath	23,619 (82.0)	596,432 (100.0)	−	−
Workers who attended:				
Physiotherapist	22,769	559,874 (93.9)	Individual	511,579 (91.4)
			Group	48,295 (8.6)
Chiropractor	1,808	23,479 (3.9)	−	−
Osteopath	873	13,079 (2.2)	−	−

The prevalence of at least one physical therapy attendance was lowest in workers from Victoria (N = 6,925, 74.4%) (see Table 2). This was reflected in the binary logistic regression model with Victorian workers at significantly lower likelihood of attending any physical therapy compared to workers from the reference state of Queensland (odds ratio (OR) 0.40, 99% confidence interval (99%CI) 0.36, 0.45). While a greater proportion of workers attended physical therapy at least once in South Australia (82.7%) and Western Australia (84.8%), they were also still at significantly lower likelihood of any physical therapy attendance than workers from Queensland (South Australia OR 0.71, 99%CI 0.61, 0.82; Western Australia OR 0.80, 99%CI 0.70, 0.91).

**Table 2:** Socio-demographic characteristics of workers that attended any physical therapy

	Workers that attended physical therapy at least once	Binary logistic regression model
	N (row %)	OR (99%CI)^1^
Sample	23,619 (82.0)	–
Sex		
Male	14,927 (80.9)	1.00 (ref)^2^
Female	8,692 (83.8)	1.29 (1.17, 1.43)*
Age group		
15–25 years	2,806 (79.8)	0.74 (0.65, 0.85)*
26–35 years	5,609 (83.7)	1.00 (0.88, 1.12)
36–45 years	6,297 (83.5)	1.00 (ref)
46–55 years	5,865 (80.8)	0.83 (0.74, 0.93)*
56 + years	3,042 (79.9)	0.81 (0.71, 0.93)*
Occupation		
Laborers	6,185 (80.2)	1.00 (ref)
Clerical and administrative workers	699 (84.1)	1.09 (0.84, 1.43)
Community and personal service workers	4,477 (81.7)	0.98 (0.86, 1.12)
Machinery operators and drivers	4,200 (82.5)	1.25 (1.11, 1.43)*
Managers	925 (83.3)	1.31 (1.05, 1.66)*
Professionals	2,032 (84.7)	1.19 (1.00, 1.42)
Sales workers	931 (84.3)	1.21 (0.96, 1.54)
Technicians and trades workers	4,169 (81.8)	1.17 (1.03, 1.33)*
Missing (n < 5)^3^	– (−)	–
Jurisdiction		
Queensland	8,684 (87.0)	1.00 (ref)
Victoria	6,925 (74.4)	0.40 (0.36, 0.45)*
South Australia	2,902 (82.7)	0.71 (0.61, 0.82)*
Western Australia	5,108 (84.8)	0.80 (0.70, 0.91)*
Socioeconomic status		
Most advantaged quintile	3,691 (84.0)	1.09 (0.97, 1.24)
Second to fourth quintiles	15,304 (82.0)	1.00 (ref)
Most disadvantaged quintile	3,556 (78.9)	0.86 (0.77, 0.96)*
Missing (n = 1,261)	1,068 (84.7)	–
Remoteness		
Major cities of Australia	16,012 (83.3)	1.00 (ref)
Regional Australia	6,234 (79.1)	0.75 (0.69, 0.83)*
Remote Australia	303 (66.3)	0.31 (0.24, 0.40)*
Missing (n = 1,264)	1,070 (84.7)	–

^*^p < 0.01

^1^Odds ratio (95% confidence interval)

^2^Reference category

^3^Cells suppressed

Females were at significantly greater likelihood of any physical therapy attendance than males (OR 1.29, 99%CI 1.17, 1.43). Workers from socioeconomically disadvantaged areas (OR 0.86, 99%CI 0.77, 0.96) and regional (OR 0.75, 99%CI 0.69, 0.83) and remote areas (OR 0.31, 99%CI 0.24, 0.40) were at significantly lower likelihood of attending any physical therapy.

Among workers that attended physical therapy, most only attended physiotherapists (N = 21,036, 89.1%), with 2.2% attending only chiropractors (N = 528), and 1.3% osteopaths (N = 296) (see Table 3). A total of 1,760 (7.5%) workers attended more than one type of therapist. The most substantial differences between these groups were attributable to worker jurisdiction. The highest proportion of workers who attended only physiotherapy were from Queensland (94.8%), with the lowest proportion in Victoria (80.0%). The highest proportion of workers who visited multiple professions were from Victoria (N = 857, 12.4%). Inter-jurisdictional differences were reflected in the multinomial logistic regression. Workers from Victoria, South Australia, and Western Australia were at significantly greater likelihood of attending only chiropractors or multiple professions compared with workers from Queensland (see Table 3). Workers from Victoria had a significantly greater likelihood of only attending osteopaths than workers from Queensland (OR 10.41, 99%CI 6.42, 16.88).

**Table 3:** Socio-demographic characteristics of workers by group (i.e., attended only physiotherapy, only chiropractic, only osteopathy or multiple physical therapy)

	Descriptive statistics by group				Multinomial logistic regression model (physio. only is reference group)		
	Physio. only	Chiro. only	Osteo. only	Multiple	Chiro. only	Osteo. only	Multiple
	N (%)	N (%)	N (%)	N (%)	OR (99%CI)^1^	OR (99%CI)	OR (99%CI)
Sample	21,035 (89.1)	528 (2.2)	296 (1.3)	1,760 (7.5)	–	–	–
Sex							
Male	13,360 (89.5)	360 (2.4)	167 (1.1)	1,040 (7.0)	1.00 (ref)^2^	1.00 (ref)	1.00 (ref)
Female	7,675 (88.3)	168 (1.9)	129 (1.5)	720 (8.3)	0.79 (0.59, 1.06)	1.04 (0.72, 1.49)	1.18 (1.00, 1.39)*
Age group							
15–25 years	2,544 (90.7)	57 (2.0)	34 (1.2)	171 (6.1)	1.01 (0.66, 1.54)	0.99 (0.58, 1.70)	0.86 (0.67, 1.10)
26–35 years	4,955 (88.3)	109 (1.9)	65 (1.2)	480 (8.6)	0.94 (0.67, 1.33)	0.90 (0.58, 1.40)	1.20 (1.00, 1.44)
36–45 years	5,615 (89.2)	130 (2.1)	82 (1.3)	470 (7.5)	1.00 (ref)	1.00 (ref)	1.00 (ref)
46–55 years	5,199 (88.6)	144 (2.5)	77 (1.3)	445 (7.6)	1.19 (0.86, 1.64)	0.92 (0.60, 1.40)	1.00 (0.83, 1.20)
56 + years	2,722 (89.5)	88 (2.9)	38 (1.2)	194 (6.4)	1.34 (0.93, 1.93)	0.84 (0.50, 1.42)	0.82 (0.65, 1.04)
Occupation							
Laborers	5,590 (90.4)	144 (2.3)	50 (0.8)	401 (6.5)	1.00 (ref)	1.00 (ref)	1.00 (ref)
Clerical and administrative workers	623 (89.1)	12 (1.7)	7 (1.0)	57 (8.2)	0.89 (0.40, 1.98)	1.35 (0.46, 3.92)	1.36 (0.91, 2.02)
Community and personal service workers	3,997 (89.3)	88 (2.0)	73 (1.6)	319 (7.1)	0.91 (0.62, 1.33)	1.87 (1.13, 3.10)*	1.04 (0.83, 1.29)
Machinery operators and drivers	3,789 (90.2)	93 (2.2)	31 (0.7)	287 (6.8)	0.78 (0.54, 1.12)	0.81 (0.44, 1.49)	1.00 (0.80, 1.24)
Managers	777 (84.0)	26 (2.8)	28 (3.0)	94 (10.2)	1.08 (0.61, 1.90)	2.51 (1.33, 4.73)*	1.32 (0.95, 1.83)
Professionals	1,785 (87.8)	38 (1.9)	32 (1.6)	177 (8.7)	0.85 (0.52, 1.41)	1.51 (0.81, 2.84)	1.24 (0.95, 1.62)
Sales workers	800 (85.9)	20 (2.1)	18 (1.9)	93 (10.0)	0.93 (0.49, 1.79)	2.17 (1.02, 4.61)*	1.47 (1.06, 2.04)*
Technicians and trades workers	3,674 (88.1)	107 (2.6)	57 (1.4)	331 (7.9)	1.02 (0.72, 1.45)	1.63 (0.97, 2.72)	1.22 (0.99, 1.50)
Missing (n < 5)^3^	– (−)	– (−)	– (−)	– (−)			
Jurisdiction^4^							
Queensland	8,229 (94.8)	85 (1.0)	35 (0.4)	335 (3.9)	1.00 (ref)	1.00 (ref)	1.00 (ref)
Victoria	5,539 (80.0)	284 (4.1)	245 (3.5)	857 (12.4)	4.85 (3.50, 6.72)*	10.41 (6.42, 16.88)*	3.82 (3.21, 4.55)*
South Australia	2,589 (89.2)	55 (1.9)	5 (0.2)	253 (8.7)	2.23 (1.40, 3.55)*	0.23 (0.04, 1.52)	2.37 (1.87, 3.02)*
Western Australia	4,678 (91.6)	104 (2.0)	11 (0.2)	315 (6.2)	2.13 (1.44, 3.17)*	0.52 (0.20, 1.34)	1.57 (1.26, 1.96)*
Socioeconomic status							
Most advantaged quintile	3,231 (87.5)	86 (2.3)	68 (1.8)	306 (8.3)	1.05 (0.76, 1.47)	1.06 (0.73, 1.56)	1.10 (0.92, 1.33)
Second to fourth quintiles	13,599 (88.9)	358 (2.3)	207 (1.4)	1,140 (7.4)	1.00 (ref)	1.00 (ref)	1.00 (ref)
Most disadvantaged quintile	3,242 (91.2)	69 (1.9)	18 (0.5)	227 (6.4)	0.77 (0.55, 1.10)	0.43 (0.23, 0.82)*	0.81 (0.66, 0.99)*
Missing^3^	963 (90.2)	15 (1.4)	< 5	87 (8.1)	–	–	–
Remoteness							
Major cities of Australia	14,280 (89.2)	330 (2.1)	240 (1.5)	1,162 (7.3)	1.00 (ref)	1.00 (ref)	1.00 (ref)
Regional Australia	5,793 (88.6)	181 (2.8)	52 (0.8)	511 (7.8)	1.50 (1.16, 1.95)*	0.66 (0.44, 1.00)	1.24 (1.06, 1.44)*
Remote Australia^5^	– (−)	– (−)	– (−)	– (−)	2.16 (0.92, 5.11)	1.32 (0.10, 18.31)	1.57 (0.90, 2.74)
Missing	962 (89.9)	17 (1.6)	< 5	87 (8.1)			

^*^p < 0.01

^1^Odds ratio (99% confidence interval)

^2^Reference category

^3^Cells suppressed

^4^Cells perturbed at random to suppress small cell sizes

^5^Regional and remote are combined in descriptive statistics due to small cell sizes

**Fig. 1:**
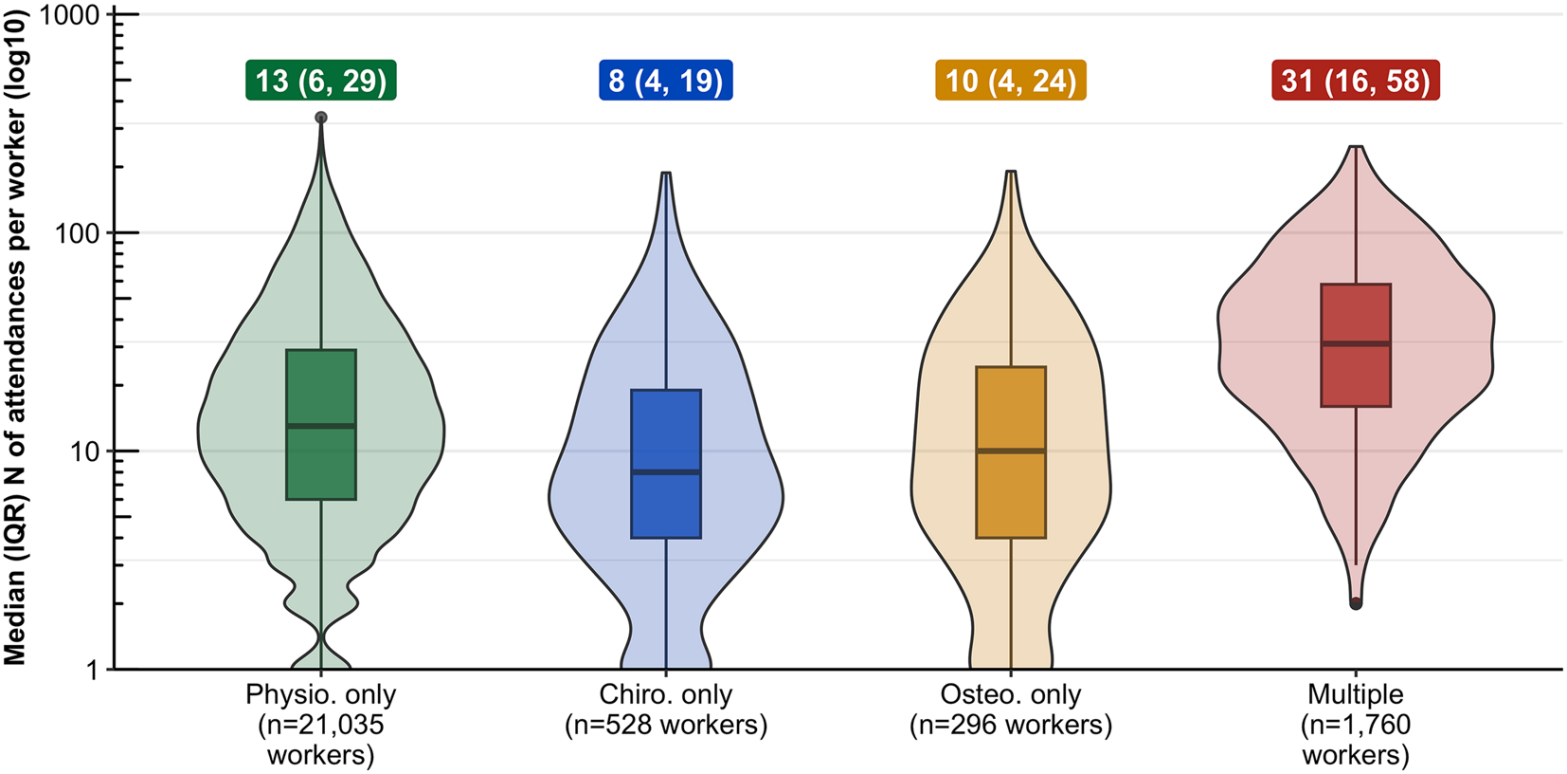
Median (IQR) and distribution of number of attendances per worker by group

The median number of attendances per worker was highest in the group who attended multiple therapists (median 31, IQR 16, 58) (see Fig. 1 and Table 4). The median number of attendances was also higher for those workers who only attended physiotherapists (13, IQR 6, 29) compared to those who only attended chiropractors (8, IQR 4, 19) or osteopaths (10, IQR 4, 24).

**Table 4:** Median and inter-quartile range (IQR) of attendances by worker group and socio-demographic characteristics

	Number of Attendances	Negative binomial regression models	
		Model 1	Model 2 (adjusted for wage replacement duration)
	Median (IQR)	IRR (99%CI)^1^	IRR (99%CI)
Attendances(s) to:			
Physiotherapy only	13.0 (6.0, 29.0)	1.00 (ref)	1.00 (ref)
Chiropractic only	8.0 (4.0, 19.0)	0.55 (0.49, 0.61)*	0.69 (0.62, 0.76)*
Osteopathy only	10.0 (4.0, 24.2)	0.52 (0.45, 0.60)*	0.63 (0.56, 0.73)*
Multiple professions	31.0 (16.0, 58.0)	1.49 (1.40, 1.58)*	1.38 (1.30, 1.46)*
Sex			
Male	13.0 (6.0, 30.0)	1.00 (ref)	1.00 (ref)
Female	15.0 (7.0, 33.0)	1.07 (1.03, 1.11)*	1.10 (1.06, 1.14)*
Age group			
15–25 years	11.0 (5.0, 24.8)	0.80 (0.76, 0.85)*	0.87 (0.82, 0.91)*
26–35 years	14.0 (7.0, 31.0)	0.95 (0.91, 1.00)*	1.00 (0.96, 1.04)
36–45 years	15.0 (7.0, 32.0)	1.00 (ref)	1.00 (ref)
46–55 years	15.0 (7.0, 33.0)	1.04 (0.99, 1.08)	1.02 (0.98, 1.06)
56 + years	14.0 (7.0, 30.0)	0.93 (0.89, 0.99)*	0.96 (0.91, 1.01)
Occupation			
Laborers	13.0 ( 6.0, 28.0)	1.00 (ref)	1.00 (ref)
Clerical and administrative workers	14.0 ( 7.0, 31.0)	1.05 (0.95, 1.16)	1.10 (1.00, 1.20)*
Community and personal service workers	14.0 ( 6.0, 29.0)	0.99 (0.94, 1.04)	1.07 (1.02, 1.12)*
Machinery operators and drivers	14.0 ( 6.0, 32.0)	1.04 (0.99, 1.09)	1.02 (0.98, 1.07)
Managers	17.0 ( 8.0, 42.0)	1.15 (1.05, 1.25)*	1.13 (1.05, 1.23)*
Professionals	16.0 ( 8.0, 35.0)	1.13 (1.06, 1.21)*	1.20 (1.13, 1.27)*
Sales workers	18.0 ( 9.0, 39.0)	1.15 (1.06, 1.26)*	1.20 (1.11, 1.30)*
Technicians and trades workers	14.0 ( 7.0, 32.0)	1.07 (1.02, 1.13)*	1.07 (1.02, 1.12)*
Missing (n < 5)^2^	– (−)	–	–
Jurisdiction			
Queensland	11.0 (6.0, 19.0)	1.00 (ref)	1.00 (ref)
Victoria	24.0 (9.0, 52.0)	2.40 (2.30, 2.49)*	1.72 (1.65, 1.78)*
South Australia	16.0 (6.0, 40.0)	1.98 (1.87, 2.09)*	1.49 (1.42, 1.57)*
Western Australia	14.0 (6.0, 31.0)	1.52 (1.46, 1.59)*	1.26 (1.21, 1.32)*
Socioeconomic status			
Most advantaged quintile	15.0 (7.0, 33.0)	0.93 (0.89, 0.98)*	0.99 (0.95, 1.03)
Second to fourth quintiles	14.0 (7.0, 31.0)	1.00 (ref)	1.00 (ref)
Most disadvantaged quintile	13.0 (6.0, 29.0)	0.97 (0.93, 1.02)	0.93 (0.89, 0.97)*
Missing	14.0 (6.0, 32.0)	–	–
Remoteness			
Major cities of Australia	15.0 (7.0, 34.0)	1.00 (ref)	1.00 (ref)
Regional Australia	12.0 (6.0, 24.0)	0.78 (0.75, 0.81)*	0.79 (0.76, 0.82)*
Remote Australia	8.0 (4.0, 18.0)	0.62 (0.54, 0.72)*	0.65 (0.57, 0.74)*
Missing	14.0 (6.0, 32.0)	ref	ref
Wage replacement duration (weeks)	– (−)		1.01 (1.01, 1.01)*

Model 1 adjusts for all covariates set at reference values (i.e., male sex, 36–45 years age group, laborers, claims from Queensland, second to fourth socioeconomic quintiles and major cities of Australia). Model 2 adjusts for the same covariates and also total cumulative wage replacement duration per claim, measured in weeks.

^*^p < 0.01.

^1^Incidence rate ratio (95% confidence interval).

^2^Cells suppressed.

Workers who only attended chiropractors or osteopaths had significantly fewer total attendances than workers who only attended physiotherapists in both model 1 and model 2 (see Table 4). Workers who attended multiple professions had significantly more attendances than those who only attended physiotherapists. Significant differences were also observed by occupation, with managers, professionals, sales workers, and technicians and trades workers likely to have a greater number of physical therapy attendances than laborers.

Workers from Victoria, South Australia, and Western Australia all had significantly more physical therapy attendances than workers from Queensland. There were minor differences in total physical therapy attendances due to socioeconomic status. Those from the most advantaged quintile had significantly fewer attendances if there was no adjustment for wage replacement duration. After adjusting for wage replacement duration (model 2), this relationship inverted, with workers from the most disadvantaged quintile having significantly fewer attendances. Finally, workers from regional and remote Australia had significantly fewer physical therapy attendances than those from major cities.

In model 2, longer wage replacement duration was associated with significantly more total physical therapy attendances. The mediating effect of adjusting for wage replacement duration was most apparent when comparing the types of therapist and worker jurisdiction. While remaining statistically significant, the size of these differences reduced after adjusting for wage replacement duration.

A maximum of N = 1,264 (4.4%) of the total sample and N = 1070 (4.5%) of workers who attended any physical therapy were excluded from statistical analyses due to missing data. Differences between exclusion and imputation methods for all statistical models presented in our results were small, did not change the significance of results, nor the direction of effect. We did not believe that imputation of this missing data would have a significant impact on our findings. Models that used imputation are available in the supplementary materials.

## Discussion

We identified that over 80% of Australian workers with accepted workers’ compensation claims for low back pain longer than 2 weeks attended physiotherapists, chiropractors, and/or osteopaths in the first 2 years of their claim. Nearly 90% of these workers only attended physiotherapists, approximately two percent solely attended chiropractors, and one percent solely attended osteopaths. Workers from Victoria had a significantly greater likelihood of only attending osteopaths compared to workers from Queensland. Workers who only attended chiropractors or osteopaths had significantly fewer median attendances compared with workers who only attended physiotherapists (8, 10, and 13 attendances, respectively).

The significantly lower proportion of workers attending for any physical therapy in Victoria may be due to the Victorian workers’ compensation scheme medical excess [24]. Physiotherapy, chiropractic, and osteopathy are primary care services that are easily accessible and frequently a first point-of-call for people with low back pain. Data used in this study only included attendances funded by the workers’ compensation scheme, i.e., after the $700AUD medical excess amount had passed. It is likely that some attendances for workers in Victoria were funded before the medical excess amount was exceeded and were subsequently not recorded in our data.

Differences in the likelihood of attending a given profession may have been driven by the availability of each type of profession. For example, in the last year of the study (2015), Victoria had 17.4 osteopaths per 100,000 residents, compared to 3.8 per 100,000 in Queensland, and 2.1 and 2.4 in South Australia and Western Australia, respectively [34]. The differences in rates of clinicians aligns with the results of this study. The high proportion of workers only attending physiotherapists in our sample sits in contrast to the nearly equal amount of physiotherapist and chiropractic services funded by private health insurance in Australia [19].

The average number of attendances was consistently highest in the group who attended multiple professions. It is possible that workers may have trialed a period of care with one profession before switching to another, initiating a new period of care and increasing the total number of attendances. We also identified that jurisdiction was associated with number of attendances. We have observed a similar trend in previous research, in which workers from Victoria attended general practitioner services a significantly greater number of times than workers from Queensland [32].

Service funding policies in each jurisdiction are likely to have contributed to differences in number of attendances. For example, a worker in Queensland can attend physiotherapy six times before requiring approval from the insurer for additional attendances [35]. Similar policies are present in other jurisdictions but appear to be less restrictive. A treatment management plan is required by the fifth attendance in Victoria [36], and the tenth attendances in South Australia and Western Australia [37, 38]. Services pricing may also contribute to these differences. As of 2023, a standard physiotherapy attendance was $64AUD in Victoria, compared to $97AUD in Queensland [35].

### Implications for Policy and Practice

The average number of attendances to any physical therapy service is considerably high in the context of recommended best practice care [3, 39, 40]. While we could not explore what treatments were delivered by each profession, it is difficult to envisage a logical justification for 8, 10, or 13 sessions with any of the included professions—let alone 31 with multiple. *The Lancet* series on low back pain call to action highlighted that many people with low back pain either do not seek or need to seek care, and the role of clinicians is primary to provide advice, reassurance, and education about self-management [41]. Health care professionals could also help workers to set realistic goals for return to work and ensure ongoing care is warranted. Recommended care for low back pain can also essentially be provided by any primary health care clinician.

Although not specific to low back pain, workers’ compensation schemes in Australia have adopted a single clinical framework for the delivery of health services [42]. The New South Wales workers’ compensation scheme (not included in our sample) has recently released an updated model of care for acute low back pain for public consultation, that recommends a total of four sessions all at pre-determined times within a total of 12 weeks [43]. This includes screening, triage, and education components, and can be provided by any primary health care clinician. Other workers’ compensation schemes could look to implement, and test the effectiveness of, similar standardized models.

### Strengths and Limitations

Our analysis benefited from a large sample of administrative data. We were also able to adjust for numerous potential confounders, including wage replacement duration. The array of administrative data from different jurisdictions also allowed us to highlight important policy-related differences. Several limitations should be noted. First, while administrative data enable us to identify that the sample of workers had an accepted claim for low back pain, we did not have clinical information about their condition such as pain severity scores or disability measures. To that end, while we could identify that a worker encountered a given type of profession, we could not identify what diagnostic or treatment modalities that clinician provided. Health care is also relatively accessible in Australia, and as highlighted earlier, Australians often access physiotherapists, chiropractors, and osteopaths through other funding sources such as private health insurance or out-of-pocket. Given we could only identify services funded by workers’ compensation, we may be underestimating total service utilization. Future research should closely examine the patterns of encounters with clinicians, particularly in those workers seeking care from multiple professions. Furthermore, analysis to assess the relationship between intensity and type of clinician encounter and worker outcomes (e.g., time to return to work) could also be conducted.

## Conclusion

Most Australians with workers’ compensation claims for low back pain lasting longer than 2 weeks attend for physical therapy in the first 2 years of their claim although the likelihood is less for males and workers in socioeconomically disadvantaged and regional and remote areas. Most workers see physiotherapists and comparatively more often compared with the 3% who only see chiropractors or osteopaths, and this is most explained by jurisdictional differences. Further research could examine the patterns of care within each profession and how this relates to workers’ outcomes.

## Supplementary Information

Below is the link to the electronic supplementary material.

Supplementary file1 (PDF 224 kb)

## Declarations

### Data Availability

Data used in this study were provided through data sharing agreements with individual data custodians and are not available for distribution by the authors. The R analysis code used to analyze the data is available in the supplementary materials.
