# Supplementary material for: How Much Physiotherapy, Chiropractic, and Osteopathy Care Do Compensated Australian Workers with Low Back Pain Receive? A Retrospective Cohort Study
Source: J Occup Rehabil. 2024 May 18;35(2):345–55. doi: 10.1007/s10926-024-10202-1 (PMC12089149; doi:10.1007/s10926-024-10202-1)
Supplement: Supplementary file 1 — Supplementary file1 (PDF 224 kb) [file 10926_2024_10202_MOESM1_ESM.pdf]

How much physiotherapy, chiropractic and osteopathy care do compensated Australian workers with low back pain receive? A retrospective cohort study

Michael Di Donato[1], Shannon Gray[1], Luke R. Sheehan[1], Rachelle Buchbinder[1], Ross Iles[1] and Alex Collie[1]

1. School of Public Health and Preventive Medicine, Monash University

Corresponding Author:

Dr Michael Di Donato

School of Public Health and Preventive Medicine, Monash University

553 St Kilda Road, Melbourne VIC 3000, Australia

E: michael.didonato@monash.edu

T: +61 03 9905 6417

SUPPLEMENTARY MATERIALS

Supplementary item 1 – Low back pain claim selection criteria

Supplementary Table 1. Low back pain selection criteria

TOOCS	***Eligible Codes***
Nature of injury	228 – Trauma to muscles and tendons, not elsewhere classified<br>229 – Trauma to muscles and tendons, unspecified<br>239 – Soft tissue injuries due to trauma or unknown mechanisms with insufficient information to code elsewhere<br>422 – Disc displacement, prolapse, degeneration, or hernia<br>459 – Back pain, lumbago, and sciatica<br>533 – Muscle / tendon strain (non-traumatic)
Location of injury	311 – Lower back
Mechanism of injury	Any
Agency of injury	Any

Supplementary item 2 – R packages used

Supplementary Table 2. R packages used in analysis

Package	***Version***	***Reference***
MASS	7.3.58.3	https://cran.r-project.org/package=MASS
base	4.2.2	https://cran.r-project.org/package=base
broom	1.0.3	https://cran.r-project.org/package=broom
datasets	4.2.2	https://cran.r-project.org/package=datasets
dplyr	1.1.0	https://cran.r-project.org/package=dplyr
forcats	1.0.0	https://cran.r-project.org/package=forcats
ggplot2	3.4.0	https://cran.r-project.org/package=ggplot2
grDevices	4.2.2	https://cran.r-project.org/package=grDevices
graphics	4.2.2	https://cran.r-project.org/package=graphics
knitr	1.42	https://cran.r-project.org/package=knitr
lubridate	1.9.1	https://cran.r-project.org/package=lubridate
methods	4.2.2	https://cran.r-project.org/package=methods
naniar	1.0.0	https://cran.r-project.org/package=naniar
nnet	7.3.18	https://cran.r-project.org/package=nnet
patchwork	1.1.2	https://cran.r-project.org/package=patchwork
purrr	1.0.1	https://cran.r-project.org/package=purrr
readr	2.1.3	https://cran.r-project.org/package=readr
scales	1.2.1	https://cran.r-project.org/package=scales
stats	4.2.2	https://cran.r-project.org/package=stats
stringr	1.5.0	https://cran.r-project.org/package=stringr
tibble	3.1.8	https://cran.r-project.org/package=tibble
tidyr	1.3.0	https://cran.r-project.org/package=tidyr
tidyverse	1.3.2	https://cran.r-project.org/package=tidyverse
utils	4.2.2	https://cran.r-project.org/package=utils

Supplementary item 3 – Sensitivity analysis of imputed missing data

Supplementary Table 3. Socio-demographic characteristics of workers that attended physical therapy, with results of binary logistic regression (1) excluding missing data and (2) imputing missing data

	MISSINGS EXCLUDED	*MISSINGS IMPUTED*
	Binary logistic regression model *OR (99%CI)*[1]	***Binary logistic regression model*** *OR (99%CI)*
Sample	-	
Sex		
Male	1.00 (ref)	1.00 (ref)
Female	1.29 (1.17, 1.43)*	1.29 (1.17, 1.43)*
Age group		
15-25 years	0.74 (0.65, 0.85)*	0.75 (0.65, 0.86)*
26-35 years	1.00 (0.88, 1.12)	1.01 (0.89, 1.13)
36-45 years	1.00 (ref)	1.00 (ref)
46-55 years	0.83 (0.74, 0.93)*	0.84 (0.75, 0.94)*
56+ years	0.81 (0.71, 0.93)*	0.80 (0.70, 0.92)*
Occupation		
Labourers	1.00 (ref)	1.00 (ref)
Clerical and Administrative Workers	1.09 (0.84, 1.43)	1.11 (0.86, 1.45)
Community and Personal Service Workers	0.98 (0.86, 1.12)	0.97 (0.85, 1.10)
Machinery Operators and Drivers	1.25 (1.11, 1.43)*	1.26 (1.12, 1.43)*
Managers	1.31 (1.05, 1.66)*	1.31 (1.05, 1.64)*
Professionals	1.19 (1.00, 1.42)	1.18 (0.99, 1.40)
Sales Workers	1.21 (0.96, 1.54)	1.22 (0.97, 1.54)
Technicians and Trades Workers	1.17 (1.03, 1.33)*	1.16 (1.03, 1.32)*
Jurisdiction		
Queensland	1.00 (ref)	1.00 (ref)
Victoria	0.40 (0.36, 0.45)*	0.41 (0.37, 0.45)*
South Australia	0.71 (0.61, 0.82)*	0.72 (0.62, 0.83)*
Western Australia	0.80 (0.70, 0.91)*	0.80 (0.71, 0.91)*
Socioeconomic status		
Most advantaged quintile	1.09 (0.97, 1.24)	1.09 (0.97, 1.23)
Second to fourth quintiles	1.00 (ref)	1.00 (ref)
Most disadvantaged quintile	0.86 (0.77, 0.96)*	0.87 (0.78, 0.97)*
Remoteness		
Major Cities of Australia	1.00 (ref)	1.00 (ref)
Regional Australia	0.75 (0.69, 0.83)*	0.75 (0.69, 0.82)*
Remote Australia	0.31 (0.24, 0.40)*	0.31 (0.24, 0.41)*

Supplementary Table 4. Socio-demographic characteristics of workers by group (i.e., attended only physiotherapy, only chiropractic, only osteopathy or multiple physical therapy professions), with results of multinomial logistic regression (1) excluding missing data and (2) imputing missing data

	MISSINGS EXCLUDED			***MISSINGS IMPUTED***		
	Multinomial logistic regression model (physio. only is reference group)			***Multinomial logistic regression model (physio. only is reference group)***		
	Chiro. only	***Osteo. only***	***Multiple***	***Chiro. only***	***Osteo. only***	***Multiple***
	OR (99%CI)[1]	*OR (99%CI)*	*OR (99%CI)*	*OR (99%CI)*[1]	*OR (99%CI)*	*OR (99%CI)*
Sample	-	-	-			
Sex						
Male	1.00 (ref)[2]	1.00 (ref)	1.00 (ref)	1.00 (ref)[2]	1.00 (ref)	1.00 (ref)
Female	0.79 (0.59, 1.06)	1.04 (0.72, 1.49)	1.18 (1.00, 1.39)*	0.81 (0.61, 1.09)	1.05 (0.73, 1.51)	1.20 (1.02, 1.41)*
Age group						
15-25 years	1.01 (0.66, 1.54)	0.99 (0.58, 1.70)	0.86 (0.67, 1.10)	1.03 (0.68, 1.56)	0.99 (0.57, 1.69)	0.84 (0.66, 1.07)
26-35 years	0.94 (0.67, 1.33)	0.90 (0.58, 1.40)	1.20 (1.00, 1.44)	0.96 (0.68, 1.35)	0.92 (0.59, 1.43)	1.19 (1.00, 1.42)
36-45 years	1.00 (ref)	1.00 (ref)	1.00 (ref)	1.00 (ref)	1.00 (ref)	1.00 (ref)
46-55 years	1.19 (0.86, 1.64)	0.92 (0.60, 1.40)	1.00 (0.83, 1.20)	1.20 (0.87, 1.65)	0.94 (0.62, 1.43)	0.98 (0.82, 1.18)
56+ years	1.34 (0.93, 1.93)	0.84 (0.50, 1.42)	0.82 (0.65, 1.04)	1.34 (0.93, 1.93)	0.87 (0.52, 1.46)	0.81 (0.65, 1.03)
Occupation						
Labourers	1.00 (ref)	1.00 (ref)	1.00 (ref)	1.00 (ref)	1.00 (ref)	1.00 (ref)
Clerical and Administrative Workers	0.89 (0.40, 1.98)	1.35 (0.46, 3.92)	1.36 (0.91, 2.02)	0.86 (0.39, 1.90)	1.33 (0.46, 3.86)	1.30 (0.88, 1.92)
Community and Personal Service Workers	0.91 (0.62, 1.33)	1.87 (1.13, 3.10)*	1.04 (0.83, 1.29)	0.91 (0.62, 1.32)	1.87 (1.13, 3.09)*	1.03 (0.83, 1.28)
Machinery Operators and Drivers	0.78 (0.54, 1.12)	0.81 (0.44, 1.49)	1.00 (0.80, 1.24)	0.83 (0.58, 1.19)	0.86 (0.47, 1.57)	1.00 (0.81, 1.24)
Managers	1.08 (0.61, 1.90)	2.51 (1.33, 4.73)*	1.32 (0.95, 1.83)	1.05 (0.60, 1.86)	2.50 (1.33, 4.70)*	1.33 (0.97, 1.83)
Professionals	0.85 (0.52, 1.41)	1.51 (0.81, 2.84)	1.24 (0.95, 1.62)	0.86 (0.52, 1.42)	1.56 (0.84, 2.91)	1.25 (0.97, 1.62)
Sales Workers	0.93 (0.49, 1.79)	2.17 (1.02, 4.61)*	1.47 (1.06, 2.04)*	0.96 (0.51, 1.81)	2.28 (1.09, 4.78)*	1.40 (1.01, 1.93)*

Technicians and Trades Workers	1.02 (0.72, 1.45)	1.63 (0.97, 2.72)	1.22 (0.99, 1.50)	1.04 (0.74, 1.46)	1.63 (0.97, 2.72)	1.22 (0.99, 1.50)
Jurisdiction						
Queensland	1.00 (ref)	1.00 (ref)	1.00 (ref)	1.00 (ref)[2]	1.00 (ref)	1.00 (ref)
Victoria	4.85 (3.50, 6.72)*	10.41 (6.42, 16.88)*	3.82 (3.21, 4.55)*	4.86 (3.52, 6.71)*	10.60 (6.54, 17.18)*	3.82 (3.21, 4.54)*
South Australia	2.23 (1.40, 3.55)*	0.23 (0.04, 1.52)	2.37 (1.87, 3.02)*	2.22 (1.42, 3.47)*	0.21 (0.03, 1.37)	2.53 (2.02, 3.17)*
Western Australia	2.13 (1.44, 3.17)*	0.52 (0.20, 1.34)	1.57 (1.26, 1.96)*	2.03 (1.38, 2.99)*	0.63 (0.27, 1.48)	1.61 (1.30, 1.99)*
Socioeconomic status						
Most advantaged quintile	1.05 (0.76, 1.47)	1.06 (0.73, 1.56)	1.10 (0.92, 1.33)	1.08 (0.78, 1.49)	1.08 (0.74, 1.58)	1.10 (0.92, 1.31)
Second to fourth quintiles	1.00 (ref)	1.00 (ref)	1.00 (ref)	1.00 (ref)[2]	1.00 (ref)	1.00 (ref)
Most disadvantaged quintile	0.77 (0.55, 1.10)	0.43 (0.23, 0.82) *	0.81 (0.66, 0.99) *	0.79 (0.56, 1.12)	0.43 (0.23, 0.82)*	0.82 (0.68, 1.00)*
Remoteness						
Major Cities of Australia	1.00 (ref)	1.00 (ref)	1.00 (ref)	1.00 (ref)[2]	1.00 (ref)	1.00 (ref)
Regional Australia	1.50 (1.16, 1.95)*	0.66 (0.44, 1.00)	1.24 (1.06, 1.44)*	1.48 (1.15, 1.92)*	0.65 (0.43, 0.99)*	1.24 (1.07, 1.44)*
Remote Australia	2.16 (0.92, 5.11)	1.32 (0.10, 18.31)	1.57 (0.90, 2.74)	2.01 (0.86, 4.75)	1.15 (0.08, 15.78)	1.51 (0.89, 2.58)

Supplementary Table 5. Number of attendances by worker group and socio-demographic characteristics, with results of negative binomial regression (1) excluding missing data and (2) imputing missing data

	MISSINGS EXCLUDED		***MISSINGS IMPUTED***	
	Model 1	***Model 2 (adjusted for wage replacement duration)***	***Model 1***	***Model 2 (adjusted for wage replacement duration)***
	IRR (99%CI)[1]	*IRR (99%CI)*	*IRR (99%CI)*[1]	*IRR (99%CI)*
Attendances(s) to:				
Physiotherapy only	1.00 (ref)	1.00 (ref)	1.00 (ref)	1.00 (ref)
Chiropractic only	0.55 (0.49, 0.61)*	0.69 (0.62, 0.76)*	0.55 (0.49, 0.61)*	0.68 (0.62, 0.75)*
Osteopathy only	0.52 (0.45, 0.60)*	0.63 (0.56, 0.73)*	0.52 (0.45, 0.60)*	0.64 (0.56, 0.73)*
Multiple professions	1.49 (1.40, 1.58)*	1.38 (1.30, 1.46)*	1.52 (1.43, 1.61)*	1.39 (1.32, 1.47)*
Sex				
Male	1.00 (ref)	1.00 (ref)	1.00 (ref)	1.00 (ref)
Female	1.07 (1.03, 1.11)*	1.10 (1.06, 1.14)*	1.07 (1.03, 1.12)*	1.10 (1.07, 1.14)*
Age group				
15-25 years	0.80 (0.76, 0.85)*	0.87 (0.82, 0.91)*	0.80 (0.76, 0.84)*	0.86 (0.82, 0.91)*
26-35 years	0.95 (0.91, 1.00)*	1.00 (0.96, 1.04)	0.95 (0.91, 0.99)*	0.99 (0.95, 1.03)
36-45 years	1.00 (ref)	1.00 (ref)	1.00 (ref)	1.00 (ref)
46-55 years	1.04 (0.99, 1.08)	1.02 (0.98, 1.06)	1.03 (0.99, 1.07)	1.01 (0.97, 1.05)
56+ years	0.93 (0.89, 0.99)*	0.96 (0.91, 1.01)	0.94 (0.89, 0.99)*	0.96 (0.91, 1.01)
Occupation				
Labourers	1.00 (ref)	1.00 (ref)	1.00 (ref)	1.00 (ref)
Clerical and Administrative Workers	1.05 (0.95, 1.16)	1.10 (1.00, 1.20)*	1.04 (0.95, 1.15)	1.09 (1.00, 1.19)*
Community and Personal Service Workers	0.99 (0.94, 1.04)	1.07 (1.02, 1.12)*	0.98 (0.94, 1.03)	1.07 (1.02, 1.11)*
Machinery Operators and Drivers	1.04 (0.99, 1.09)	1.02 (0.98, 1.07)	1.04 (0.99, 1.09)	1.02 (0.98, 1.07)

Managers	1.15 (1.05, 1.25)*	1.13 (1.05, 1.23)*	1.15 (1.06, 1.26)*	1.15 (1.06, 1.24)*
Professionals	1.13 (1.06, 1.21)*	1.20 (1.13, 1.27)*	1.12 (1.05, 1.20)*	1.19 (1.13, 1.27)*
Sales Workers	1.15 (1.06, 1.26)*	1.20 (1.11, 1.30)*	1.14 (1.05, 1.25)*	1.19 (1.10, 1.29)*
Technicians and Trades Workers	1.07 (1.02, 1.13)*	1.07 (1.02, 1.12)*	1.06 (1.01, 1.11)*	1.07 (1.02, 1.11)*
Jurisdiction				
Queensland	1.00 (ref)	1.00 (ref)	1.00 (ref)	1.00 (ref)
Victoria	2.40 (2.30, 2.49)*	1.72 (1.65, 1.78)*	2.39 (2.29, 2.48)*	1.71 (1.65, 1.78)*
South Australia	1.98 (1.87, 2.09)*	1.49 (1.42, 1.57)*	2.00 (1.90, 2.11)*	1.48 (1.41, 1.55)*
Western Australia	1.52 (1.46, 1.59)*	1.26 (1.21, 1.32)*	1.52 (1.45, 1.58)*	1.26 (1.21, 1.31)*
Socioeconomic status				
Most advantaged quintile	0.93 (0.89, 0.98)*	0.99 (0.95, 1.03)	0.93 (0.89, 0.98)*	0.99 (0.95, 1.03)
Second to fourth quintiles	1.00 (ref)	1.00 (ref)	1.00 (ref)	1.00 (ref)
Most disadvantaged quintile	0.97 (0.93, 1.02)	0.93 (0.89, 0.97)*	0.98 (0.94, 1.03)	0.93 (0.89, 0.96)*
Remoteness				
Major Cities of Australia	1.00 (ref)	1.00 (ref)	1.00 (ref)	1.00 (ref)
Regional Australia	0.78 (0.75, 0.81)*	0.79 (0.76, 0.82)*	0.79 (0.76, 0.82)*	0.80 (0.77, 0.82)*
Remote Australia	0.62 (0.54, 0.72)*	0.65 (0.57, 0.74)*	0.62 (0.55, 0.72)*	0.66 (0.59, 0.75)*
Wage replacement duration (weeks)		1.01 (1.01, 1.01)*	-	1.01 (1.01, 1.01)*
